# Counselling and co-opting parents to get best outcomes

**Published:** 2018

**Authors:** Subhadra Jalali, Divya Bala Krishnan

**Affiliations:** Deputy Director: Newborn Eye Health Alliance (NEHA) and Director, Quality: LV Prasad Eye Institute, Hyderabad, India.; Consultant: Srimati Kannuri Santhamma Centre for Vitreoretinal Diseases and Jasti V Ramanamma Childrens Eye Care Centre, Kallam Anji Reddy Campus, L V Prasad Eye Institute, Hyderabad, India.

**Figure:**
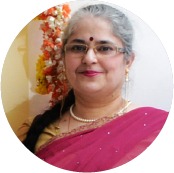
Subhadra Jalali

**Figure:**
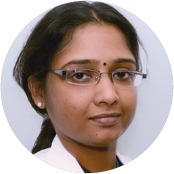
Divya Bala Krishnan

**Timely, realistic and appropriate counseling of parents and making them a part of the team at each step is a critical way to get the best vision for the baby.**

Retinopathy of prematurity is one of the leading causes of preventable blindness in preterm babies. The number of preterm deliveries is increasing. Although the survival of these preterm babies is on the rise due to better and more accessible neonatal care, the situation leads to a period of great stress to family members. This includes emotional, monetary, physical and logistical stress to those involved in giving care to the fragile baby (Figure 1).

**Figure 1:**
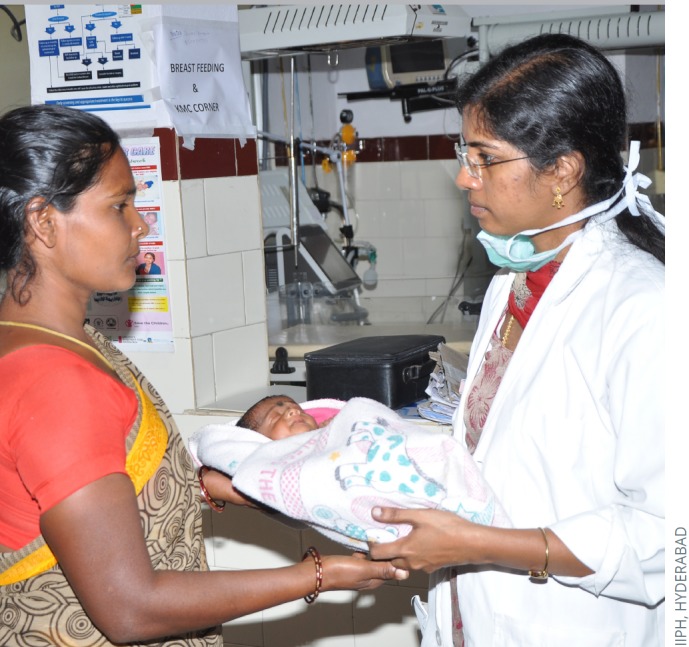
An anxious mother waiting for her baby's eye screening at Niloufer Hospital, Hyderabad, India.

In such a situation, the ophthalmology care team has to provide the best possible vision and least ocular morbidity. The main factors in preventing blindness due to ROP are timely screening and treatment, as this is a time-bound disease. The disease occurs in at-risk babies around three to four weeks after birth. A timely screening (between 20–30 days after birth) will help in detecting any sight threatening ROP. Very close follow up, sometimes every three to seven days is needed during this time which requires close coordination between all stakeholders.

Major factors that need to be tackled at various levels to prevent ROP blindness are discussed below:

## Factors responsible for delayed screening of preterm babies for ROP are:

(Table 1)

## Factors responsible for delay in treatment and non-compliance to follow up are:

- Non-availability of ophthalmologists trained in laser or surgical treatment.
- Delay in initiating treatment even after detecting treatable disease, due to financial and logistic constraints. Most often parents have to travel a long distance with babies to reach specialist doctor for treatment. The expenditure for two to three attendants who may have to accompany the baby becomes unaffordable. This is especially after having spent a major amount of money for NICU care.
- Ignorance among parents regarding the need for regular and long-term follow up and the threat to sight if the treatment is delayed.
- Unlike other diseases, the symptoms of vision loss due to ROP are manifested in the late stage of disease. Unless the parents are made aware of this, they are likely to delay the eye check-up.
- Sometimes parents do not consent to timely treatment as they are not sure of the benefits of treatment. The lack of trust in modern medicine with apprehension that some ‘experiment’ may be done on their baby are a few of the reasons.

## Care factors responsible for worsening of ROP or severe ROP:

Most factors that lead to severe forms of ROP are related to low gestational age and the type of medical care. Parents can help in caring for their infants by

- washing hands regularly,
- breast feeding on time,
- giving kangaroo mother care,
- taking good nutritious food while breastfeeding,
- taking proper care of cough and cold episodes in the baby, and
- overall positive communication while interacting with the baby.

Some of these factors may not have robust evidence in terms of ROP management but are part of good practices and low-cost additions for integrated and improved neonatal care.

## Why counsel parents?

Most of the babies, who need eye evaluation and treatment are either preterm or have significant co-morbidities. It is imperative that team members work in coordination to ensure safe and timely screening. Lack of coordination or lack of appreciation of each others' roles, or lack of faith in the management can lead to needless delays and doubts in the parents' mind.

In most cases, neither our medical training nor our mainstream ophthalmic or newborn care literature and training address this issue. Over more than a decade, we have personally tried to understand and devise ways to build the team. It has been a continuous learning and refining process and in this article we share our experiences.

**Table 1:** Myths and facts about ROP

Factor	Myth	Fact
**Sickness**	Sick babies should not be screened or treated as it can lead to death.	Sick babies can be screened and treated safely using short protocols and appropriate safety monitoring against pain, hypoglycemia, apnoea, bradycardia and hypothermia
**Care status and ROP**	Since the care provided is excellent or baby did not get any oxygen or was given only minimum oxygen, babies will not get ROP and hence no need to screen.	Blinding ROP can occur even in those preterm babies who have never received oxygen and even if they have got the best possible care. Large for gestational age babies can also be affected, because it is the prematurity itself that is the major risk factor. Hence screening is mandatory.
**Taking baby home**	After discharge the preterm babies should not be taken out of home and hence cannot go for ROP screening as they can catch infection or are too fragile to be moved out.	Babies can be taken for screening after discharge from the hospital by taking proper precautions such as wrapping baby well for warmth and infection control, feeding on time and keeping the baby close to the mother
**Appearance of eyes**	Since eyes look normal, eye check can be done later once baby gains weight.	Baby cannot express reduced or blurred vision. Serious eye problems remain hidden and are not visible in the eye till stage 5 of ROP when child has become irreversibly blind.
**Public perception**	Eye check is an unnecessary test that doctors do for money, or for research or just as an extra precaution.	ROP screening is mandatory for all at-risk preterm babies because of severe irreversible blindness in such babies that can only be prevented by timely screening and urgent treatment
**Newborn care givers' perception**	Vision development of newborns is part of neuro-developmental assessment and can be done once baby is relieved of all critical care issues.	To preserve the vision of newborns ROP care is integral to the critical management of newborns and cannot be postponed to the phase of neuro-developmental assessments.
**Information to parents about ROP**	Writing that ‘ROP evaluation to be done’ in the discharge summary and informing ‘get eye check also done’ is enough to ensure compliance by parents to timely ROP screening.	Detailed awareness creation by brochures, wall posters, videos on TV in waiting lounges and proper verbal explanation to each parent about need for timely ROP screening and asking for the ROP screening report at each follow-up visit can improve compliance.
**Parents role**	Parents are ignorant and are on one side of the management and should give the written or implied consent but are not part of the core team that provides the medical care. Medical care teams ‘know everything’ and are on the other side.	Best outcomes are achieved when parents are co-opted to be included in the core team of providing ROP management. Strong communication system and appreciation of each others' roles helps in overcoming many challenges.

We have experienced that despite all the above-mentioned factors, if we educate the parents about the importance of regular check-ups, then every parent will make the effort to prioritise it over their personal and family matters.^1^,^2^ Health education literature also indicates that parent education can play a significant role in getting them to prioritise health issues.

**Figure 2:**
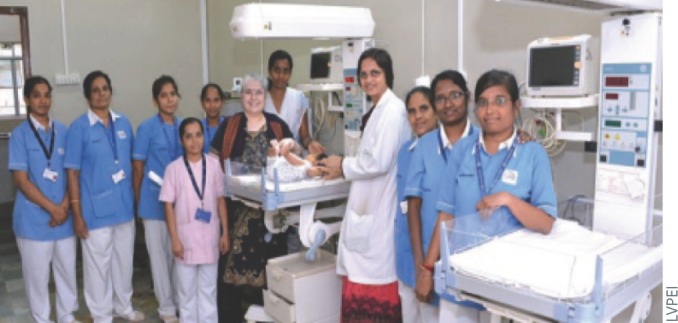
Mother, team of doctors and nurses partner to care for baby with ROP.

Timely, realistic and appropriate counseling of parents and making them a part of the team (Figure 2) at each step is a critical way to get the best vision for the baby. If a screening facility is not available in the NICU, the parents should be motivated to take the babies to the nearest available eye hospital. The display of posters and videos of babies showing the sequelae of delayed treatment will help to create the awareness. Connecting parents of babies affected with ROP, ones who had timely treatment and ones who had delayed treatment will help to gain the trust of parents.

## How to co-opt parents into the team and make the processes more robust?

### The team

The team has to include an ophthalmologist, a paediatrician/neonatologist, a duty doctor (fellow/resident), nursing staff, parents and sometimes extended family like grandparents/uncles etc and the receptionist/administrator at the neonatology and ophthalmology clinics.

### Roles

Each person in the team has to contribute to a safe, effective and efficient service for the baby. Communication, protocols and procedures must be well-defined and standardised so that everyone is on the same page.

The neonatologist is the first point of contact with the baby and family. He/she has to take complete ownership for the smooth transition of the baby through the process of ROP screening and follow-up until the baby is safely discharged from any risk of blindness. This has to be done while taking care of other critical day-to-day issues of a fragile baby. Parents should be counseled that all delivery and post-natal treatment related documents/discharge summary are essential during ROP management.

Delay in laser surgery even by a day can cause irreversible damage. It is critical to counsel parents about the urgency of the situation. Studies have shown that if doctor sits down and talks, the perception is more reassuring to the family, than if the doctor stands while talking, even if the same things are said!

With the help of diagrams/photos/images the urgency of the situation and eye condition needs to be explained in simple terms in a language that is understood by parents. The small risk of apnoea/bradycardia or even rarer need for possible ventilation, must be explained. The language used here is very critical. No negative words should be used. For example, words like “damage” or “failure” can be replaced by positive sounding statements such as “may not be as good as we want to achieve” or “we may not succeed in our attempt but can definitely try and do our best.”

Making parents or extended families part of your team is the next crucial step. Stressing on collectively taking care of the baby helps.

### Tips for speaking to parents

- The baby is fragile and you know this. The eye condition needs immediate treatment.
- What we need to do is to take care of your baby and the eyes as a team. We-you and I-are responsible for this baby.
- Please do not ask me to take all the responsibility and I won't ask you either
- Time is of essence: keep your documents ready, do not delay the surgery.
- All those who are handling the baby must wash their hands regularly
- Please feed the baby on time and continue to give kangaroo care
- Your baby will need surgery, and like all surgery there are risks. My team has a good safety record and you are in safe hands
- If you have any questions about the surgery, please don't hesitate to ask. You can always contact me by phone or email later too.
- The surgery is a difficult one. If it succeeds, I will celebrate with you. If it does not, I will be with you during that difficult time as well.

Body language and tone are critical in such conver-sations. Ensure everyone is sitting comfortably. Talk to the parents and hold the baby's hand while maintaining eye contact with the parents. The parents need to understand the gravity of the situation. They also need to be assured that care is at hand.

Give the family few minutes to digest the information. They are sure to come back to you with some questions. Take time to answer them. Our body language must depict that we have ample time to answer their queries. Generally they have one or two small queries only but our attitude should convey our empathy. If they have more questions, then direct them to a counselor who can explain in more detail. Sometimes writing down all their questions also helps. Sharing your email address or phone numbers can save time during a busy OPD. In most cases ROP screening and treatment sessions run smoothly and safely. Once ROP regresses and baby starts to see, it is time for the whole ‘team’ to relax and rejoice!

## Counselling during adverse events

In a rare event of death or moving the baby to a ventilator, the ophthalmologist and the neonatologist should stand together with the parents to share their grief in those difficult moments. The surgeon along with other professionals must note down all information with proper time and chronology of events. He/she must also be the one to speak to the parents so no confusing information or versions of events are communicated. The team of surgeons, neonatologist, anesthetists, and parents must work together with deep understanding of each other's critical roles. This is the key to success and overcoming challenges.

### Steps for enhanced team work

- Place information posters in local languages about ROP in waiting areas of SNCUs/NICUs.
- Give information brochures/handouts in local languages to parents at the time of admission. This must include space to write dates of ROP screening.
- If the baby is not going to be discharged soon, ensure that screening is done within 20–30 days of birth. If the baby is getting discharged early then screening must be done before discharge. This is to ensure that parents are sensitised about need for next follow-up.
- Clearly mention the exact date, time, place and doctor's name to be contacted for ROP screening.
- At time of discharge re-emphasise the written information orally by a member of the NICU/SNCU hospital team. Underline important statements on the information sheet so that it is not missed by parents.
- If baby is not going to return for a follow-up, give clear written instructions to the next doctor about prompt and timely screening. Emphasise the date and the high risk of irreversible blindness if instructions are not followed.
- Customising measures to specifically address the logistical and monetary challenges especially of parents from rural communities can enhance follow-up and compliance.^2^
- Counselling and creating awareness helps to provide a tight net of vision loss prevention around the baby.
